# Pharmacological Inhibition of Endogenous Hydrogen Sulfide Production Slows Bladder Cancer Progression in an Intravesical Murine Model

**DOI:** 10.3390/ph17091212

**Published:** 2024-09-14

**Authors:** Sydney Relouw, George J. Dugbartey, Patrick McLeod, Natasha N. Knier, Francisco Martinez Santiesteban, Paula J. Foster, Heather-Anne Cadieux-Pitre, Nicole M. Hague, Jenna Caine, Kaitlin Belletti, Sally Major, Caroline O’Neil, Manal Y. Gabril, Madeleine Moussa, Melissa J. Huynh, S.M. Mansour Haeryfar, Alp Sener

**Affiliations:** 1 Department of Microbiology & Immunology, Western University, London, ON N6A 5C1, Canada; 2 Matthew Mailing Center for Translational Transplant Studies, Western University, London Health Sciences Center, London, ON N6A 5A5, Canada; gdugbart@uwo.ca (G.J.D.);; 3 Department of Surgery, Western University, London, ON N6A 5C1, Canada; 4 Department of Pharmacology & Toxicology, School of Pharmacy, College of Health Sciences, University of Ghana, Legon, Accra P.O. Box LG43, Ghana; 5 Department of Physiology & Pharmacology, Accra College of Medicine, East Legon, Accra P.O. Box CT9828, Ghana; 6 Department of Medical Biophysics, Western University, London, ON N6A 5C1, Canada; 7 Robarts Research Institute, Western University, London, ON N6A 3K7, Canada; 8 Department of Animal Care & Veterinary Services, Western University, London, ON N6A 5C1, Canada; 9 Department of Pathology and Laboratory Medicine, Western University, London, ON N6A 5C1, Canada; manal.gabril@lhsc.on.ca (M.Y.G.);; 10 Lawson Health Research Institute, London, ON N6C 2R5, Canada; 11 Department of Medicine, Division of Clinical Immunology & Allergy, Western University, London, ON N6A 5C1, Canada; 12 Department of Oncology, Western University, London, ON N6A 5C1, Canada

**Keywords:** bladder cancer (BC), gemcitabine, hydrogen sulfide (H_2_S), intravesical administration, magnetic resonance imaging, propargylglycine (PAG), apoptosis, tumor progression, cystathionine γ-lyase (CSE)

## Abstract

Present bladder cancer therapies have relatively limited therapeutic impact and account for one of the highest lifetime treatment costs per patient. Therefore, there is an urgent need to explore novel and optimized treatment strategies. The present study investigated the effects of inhibiting endogenous hydrogen sulfide (H_2_S) production on bladder cell viability and in vivo tumor progression. We targeted the H_2_S-producing enzyme, cystathionine γ-lyase, in 5637 cells using propargylglycine (H_2_S inhibitor) and performed cytofluorimetric analysis to evaluate cell viability. We then tested the efficacy of propargylglycine alone or in combination with gemcitabine (conventional chemotherapy) in an intravesical murine model of bladder cancer. Magnetic resonance imaging and immunohistochemical staining for cell proliferation, apoptosis, immune-cell infiltration, and neovascularization were performed to evaluate tumor response. Compared to control conditions or cohorts, propargylglycine administration significantly attenuated bladder cancer cell viability in vitro (*p* < 0.0001) and tumor growth (*p* < 0.002) and invasion in vivo. Furthermore, propargylglycine enhanced the anti-cancer effects of gemcitabine, resulting in tumor regression (*p* < 0.0001). Moreover, propargylglycine induced cleaved PARP-1-activated apoptosis (*p* < 0.05), as well as intratumoral CD8^+^ T cell (*p* < 0.05) and F4/80^+^ macrophage (*p* < 0.002) infiltration. Propargylglycine also reduced intratumoral neovascularization (*p* < 0.0001) and cell proliferation (*p* < 0.0002). Importantly, the pro-apoptotic and anti-neovascularization effects of gemcitabine were enhanced by propargylglycine co-administration. Our findings suggest that inhibition of endogenous H_2_S production can be protective against bladder cancer by enhancing the chemotherapeutic action of gemcitabine and may be a novel pharmacological target and approach for improved bladder cancer diagnosis and treatments in the future.

## 1. Introduction

Bladder cancer (BC) is the 10th most common malignancy worldwide, with almost 600,000 new cases and over 200,000 associated deaths occurring in 2020 alone [1]. It is ranked the 13th most deadly cancer globally, the 6th most common cancer in men, and the 17th most prevalent cancer in women [2,3]. At the time of diagnosis, 75% of patients have non-muscle-invasive BC (NMIBC), and 25% have muscle-invasive BC (MIBC) [4]. Despite most cases being superficial, there is a 78% chance of recurrence and a 45% chance of progression after five years [5]. These high rates paired with lengthy treatment times and associated therapeutic complications give BC one of the highest lifetime treatment costs per patient compared to other malignancies [6,7,8,9,10]. Thus, there is an imperative need for the investigation of novel BC therapies.

Hydrogen sulfide (H_2_S) is a gaseous signaling molecule endogenously produced in all mammalian cells. It is synthesized through the reverse transsulfuration pathway by three independent enzymes, namely cystathionine β-synthase (CBS), cystathionine γ-lyase (CSE), and 3-mercaptopyruvate sulftransferase (3-MST) [11,12,13]. CBS, a cytosolic enzyme, catalyzes the condensation of homocysteine with cysteine to produce cystathionine and H_2_S [11]. CSE, also a cytosolic enzyme, catalyzes the conversion of L-cysteine to thiocysteine, which breaks down into pyruvate and H_2_S nonenzymatically [12]. After cysteine is converted to mercaptopyruvate, the enzyme 3-MST, localized in the mitochondria, further breaks the substrate down into H_2_S and pyruvate [13]. At physiological levels, H_2_S is cytoprotective and exerts positive bioenergetic effects. However, biological responses to H_2_S demonstrate a bimodal effect, where negligible or high concentrations of H_2_S are toxic and attenuate pathophysiologic pathways [14].

H_2_S is emerging as an oncogenic gas, whose dysregulation through alteration of the presence and activity of the H_2_S-producing enzyme, is associated with cancer progression [15]. Clinical and experimental evidence show that increased H_2_S production mediates the development and progression of cancers such as colon cancer, ovarian cancer, breast cancer, and melanoma due to an overexpression of H_2_S-producing enzymes at these locations, and that inhibition of these enzymes inhibits tumorigenic signaling pathways [15,16,17,18,19,20]. Despite these findings, the effect of H_2_S in BC is still a relatively new topic in research. The mere presence of CSE, CBS, and 3-MST had not been reported in human BC tissue until 2016 when Gai et al. [21] demonstrated H_2_S metabolism dysregulation in BC by revealing low H_2_S expression and productivity in healthy bladder tissues, moderate expression and productivity in NMIBC tissues, and high expression and productivity in MIBC tissues. A subsequent study demonstrated a potentiation of proliferation and invasion ability of BC cells following H_2_S administration, yet overexpression of CSE and CBS inhibited proliferation and promoted apoptosis of BC cells [22]. These findings suggest that dysregulation of H_2_S may also play an important role in BC progression.

Using a subcutaneous animal model of BC, Wahafu et al. [23] reported that the inhibition of endogenous H_2_S production potentiates the anti-cancer effects of cisplatin chemotherapy, a therapeutic agent that is clinically administered systemically. Although promising, a more clinically relevant model is required to validate these findings. An ideal model of BC would allow for tumors to (i) be of urothelial origin; (ii) grow within the bladder to interact with other layers of the bladder wall and receive direct exposure to intravesical therapies; and (iii) be easy to develop within a reasonable timeframe, thus yielding reproducible and reliable results [24]. According to these criteria, the N-butyl-N-(4-hydroxybutyl) nitrosamine (BBN) murine model of BC constitutes a suitable pre-clinical system with which to explore experimental interventions. Moreover, intravesical therapy is the clinical standard of care for BC. This, in combination with criterion 2, strongly suggests that this mode of treatment be utilized. The global shortage of the main intravesical therapeutic agent, Bacillus Calmette-Guerin, has necessitated the use of alternative substitutes such as gemcitabine (GEM). Therefore, the purpose of this study was to investigate the effects of inhibiting endogenous H_2_S production alone and in combination with GEM by intravesical administration on BC progression using a BBN murine model. We reported that inhibiting endogenous H_2_S production reduces tumor progression and potentiates the anti-cancer effects of GEM chemotherapy.

## 2. Results

### 2.1. CSE Gene Expression Is Upregulated under Hypoxic Conditions

**Figure 1:**
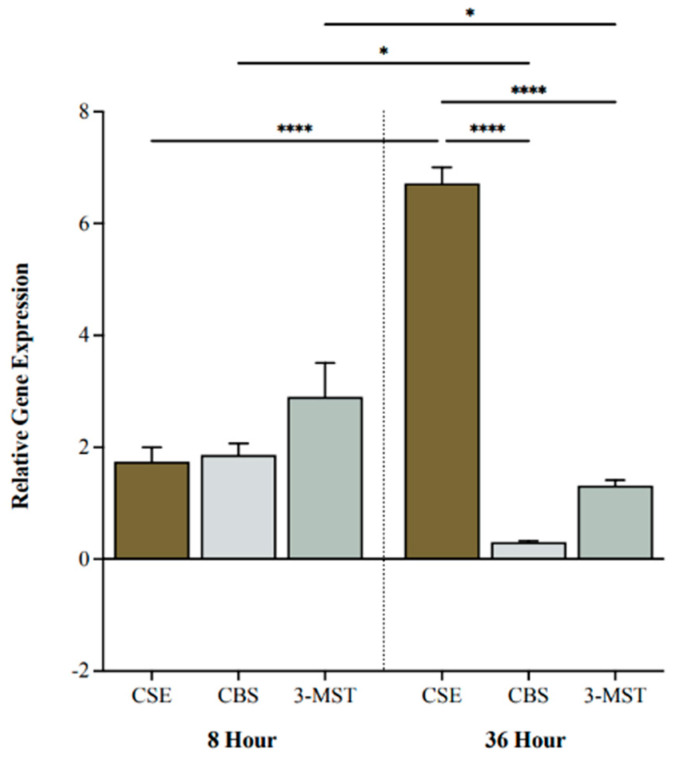
Relative gene expression of cystathionine γ-lyase (CSE), cystathionine β-synthase (CBS), and 3-mercaptopyruvate sulftransferase (3-MST) under hypoxic conditions. Quantitative PCR (qPCR) analysis of 5637 cells for CSE, CBS, and 3-MST gene expression levels after 8 and 36 h of hypoxia. Genes were normalized against β-actin, and fold changes of gene expression were compared to cells exposed to 0 h of hypoxia and calculated using the ∆∆Ct method. Data (*n* = 5) are expressed as mean ± standard error of the mean (SEM). Means were compared using two-way ANOVA followed by Tukey’s post hoc test. * *p* < 0.05, **** *p* < 0.0001.

We investigated BC cell expression levels of H_2_S-producing enzymes following 0, 8, or 36 h of hypoxia. CSE, CBS, and 3-MST were all upregulated after 8 h of hypoxia compared to 0 h (Figure 1). After 36 h, CSE was significantly more upregulated compared to CBS and 3-MST (*p* < 0.0001 and *p* < 0.0001, respectively; Figure 1). Moreover, CSE was markedly increased at 36 h compared to 8 h (*p* < 0.0001), whereas CBS and 3-MST were significantly decreased (*p* < 0.05 and *p* < 0.05, respectively; Figure 1).

### 2.2. Inhibiting CSE Activity Attenuates BC Cell Viability in the Presence of Chemotherapy

We investigated the effect of inhibiting endogenous H_2_S production on BC cell viability following treatment with the CSE inhibitor propargylglycine (PAG), the H_2_S donor sodium hydrosulfide (NaHS), and the chemotherapeutic agent GEM. PAG indirectly targets the pyridoxal 5-phosphate cofactor of CSE by sterically hindering the accessibility of the active site. NaHS directly releases H_2_S by dissociating into Na^+^ and HS^-^, which subsequently binds to H^+^.

Cell viability was assessed by staining apoptotic and necrotic cells using FITC-Annexin-V and propidium iodide. The number of stained cells was then quantified using flow cytometry.

**Figure 2:**
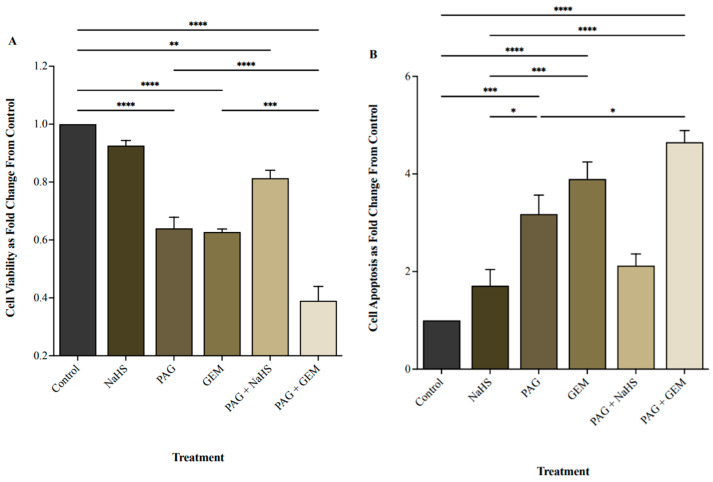
Cell viability and apoptosis following single and combination treatments with propargylglycine (PAG), sodium hydrosulfide (NaHS), and gemcitabine (GEM). (**A**) Cell viability and (**B**) apoptotic levels of 5637 cells following 8 h of hypoxia, single or combination treatments of 20 mM PAG, 100 μM NaHS, and 100 μM GEM, and an additional 24 h of hypoxia. Flow cytometry was used to quantify cell viability as the portion of cells negative for the apoptosis and necrosis markers, FITC-Annexin-V, and propidium iodide. Cell viability is represented as fold change from control cells that had undergone hypoxia without treatment. Apoptosis was quantified as the portion of cells positive for FITC-Annexin-V and negative for propidium iodide and represented as fold change from control cells that had undergone hypoxia without treatment. Data (*n* = 5) are expressed as mean ± SEM. Means were compared using one-way ANOVA followed by Tukey’s post hoc test. * *p* < 0.05, ** *p* < 0.002, *** *p* < 0.0002, **** *p* < 0.0001.

NaHS did not affect cell viability, whereas PAG and GEM markedly decreased cell viability compared to control cells (*p* < 0.0001 and *p* < 0.0001, respectively; Figure 2A). PAG and GEM in combination further reduced cell viability compared to PAG and GEM alone (*p* < 0.0002 and *p* < 0.0001, respectively), while NaHS partially restored cell viability from PAG compared to the control (*p* < 0.002, Figure 2A). Inversely, NaHS did not affect apoptosis, whereas PAG and GEM enhanced apoptosis (*p* < 0.0002 and *p* < 0.0001, respectively; Figure 2B). PAG and GEM in combination increased apoptotic cell death compared to PAG alone (*p* < 0.05), and NaHS insignificantly reduced apoptotic cell death from PAG compared to the control (Figure 2B).

### 2.3. Inhibiting CSE Activity Promotes Tumor Regression and Abrogates Invasion in the Presence of Chemotherapy

**Figure 3:**
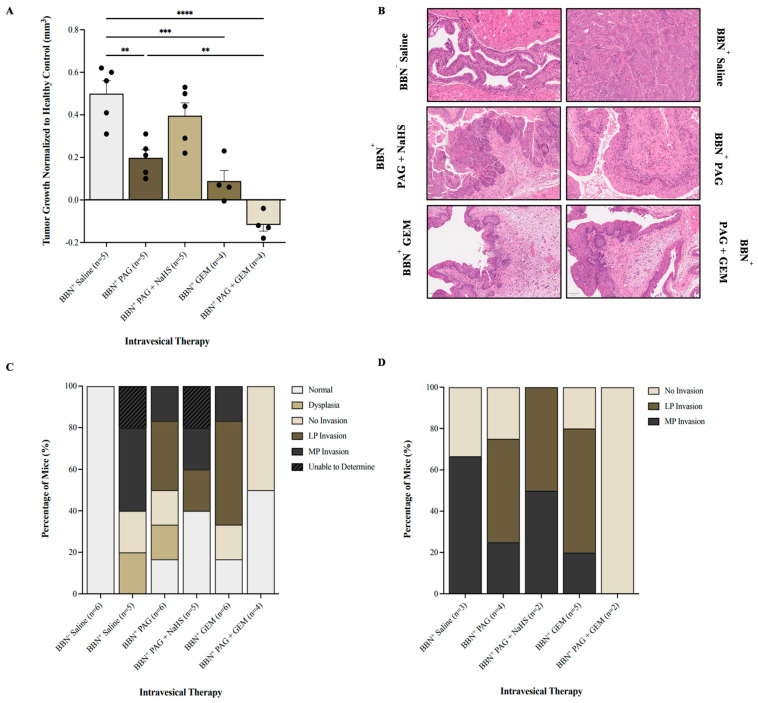
Tumor response as evaluated by magnetic resonance imaging (MRI) and cancer presence and degree of invasion following intravesical therapy. (**A**) MRI was used to assess the change in bladder wall volume, representative of tumor progression, before and after intravesical therapy of saline, PAG, NaHS, GEM, PAG + NaHS, or PAG + GEM. Changes in bladder wall volume of the N-butyl-N-(4-hydroxybutyl) nitrosamine^+^ (BBN^+^) groups were normalized to the change in bladder wall volume of the BBN^−^ group. (**B**) Representative images of hematoxylin- and eosin-stained bladder tumor tissue; 40× magnification. Scale bar represents 100 µm. (**C**) Percentage of mice within each group that had normal tissue, dysplasia, cancer with no invasion, lamina propria (LP) invasion, or muscularis propria (MP) invasion. (**D**) Percentage of mice with no invasion, LP invasion, or MP invasion among the mice that had bladder tumors. Data are expressed as mean ± SEM. Means were compared using one-way ANOVA followed by Tukey’s post hoc test. ** *p* < 0.002, *** *p* < 0.0002, **** *p* < 0.0001.

We investigated the effect of inhibiting endogenous H_2_S production on BC progression using magnetic resonance imaging (MRI; Supplementary Figure S1) and histology. It is important to note that seven MRI visuals were unable to be analyzed due to poor resolution, leading to a discrepancy in the number of mice treated and those analyzed. In comparison with the BBN^+^ saline group, monotherapy with PAG and GEM attenuated tumor growth (*p* < 0.002 and *p* < 0.0002, respectively) and further attenuated with PAG and GEM combination therapy (*p* < 0.0001, Figure 3A). Combination therapy with PAG and NaHS abrogated the anti-cancer effects of PAG, partially recovering tumor growth compared to the BBN^+^ saline group (*p* > 0.05, Figure 3A).

Our hematoxylin and eosin staining showed that the BBN^−^ saline group had 100% normal tissue while the BBN^+^ saline and BBN^+^ PAG groups had 0% and 16.7% normal tissue, respectively (Figure 3C). The BBN^+^ PAG + NaHS group had 40% normal tissue, whereas the BBN^+^ GEM and BBN^+^ PAG + GEM groups had 16.7% and 50% normal tissue, respectively (Figure 3C). Among the mice that had bladder tumors, the BBN^+^ saline group had 33% no invasion and 67% muscularis propria (MP) invasion (Figure 3D). The BBN^+^ PAG group had 30% no invasion, 40% lamina propria (LP) invasion, and 30% MP invasion, while the BBN^+^ PAG + NaHS group had 50% LP invasion and 50% MP invasion (Figure 3D). The BBN^+^ GEM group had 20% no invasion, 60% LP invasion, and 20% MP invasion, whereas the BBN^+^ PAG + GEM group had 100% no invasion (Figure 3D).

### 2.4. Inhibiting CSE Activity Induces Bladder Tumor Apoptosis, Attenuates Neovascularization and Proliferation, Alters Bladder Tumor Immune Response, and Enhances Pro-Apoptotic and Anti-Neovascularization Effects of Chemotherapy

**Figure 4:**
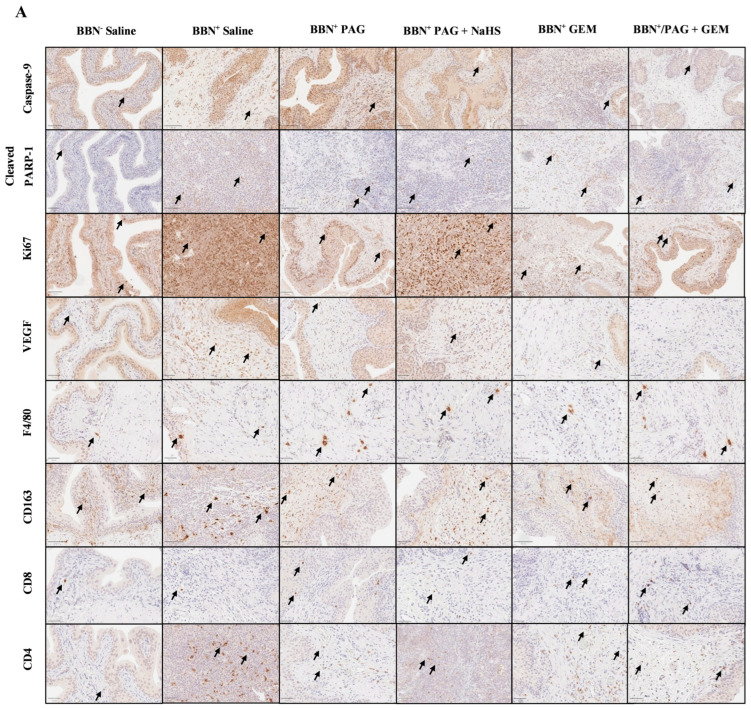

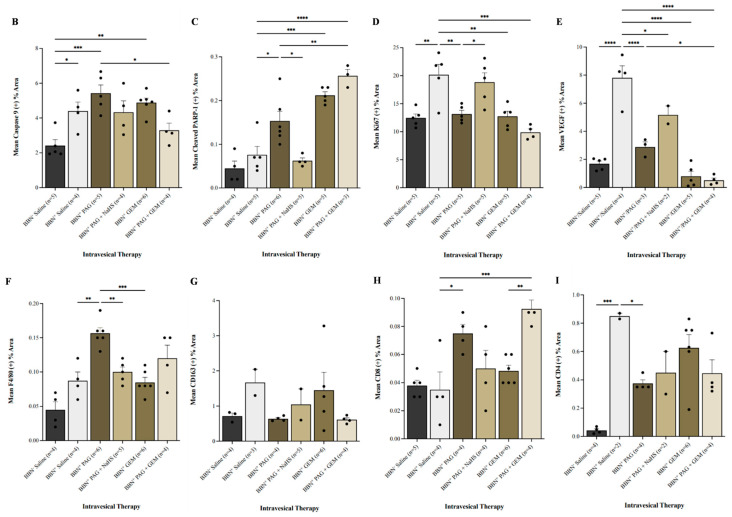
Immunohistochemical (IHC) staining of bladder tumors for cell proliferation, apoptosis, immune-cell infiltration, and neovascularization following intravesical therapy. (**A**) Representative images of bladder tumor samples stained for caspase-9, cleaved PARP-1, Ki67, VEGF, F4/80, CD163, CD8, and CD4 after intravesical therapy of saline, PAG, NaHS, GEM, PAG + NaHS or PAG + GEM; 40× magnification. Scale bar represents 100 µm. Arrows point to positively stained areas (**B**–**I**) Corresponding digital analyses show percent area of sections positive for caspase-9, cleaved PARP-1, Ki67, VEGF, F4/80, CD163, CD8, and CD4. Data are expressed as mean ± SEM. Means were compared using one-way ANOVA followed by Tukey’s post-hoc test. * *p* < 0.05, ** *p* < 0.002, *** *p* < 0.0002, **** *p* < 0.0001.

We investigated the mechanisms by which inhibiting endogenous H_2_S production attenuates bladder tumor progression and enhances the anti-cancer effects of GEM using immunohistochemical (IHC) staining for markers of apoptosis (caspase-9 and poly [ADP-ribose] polymerase 1 (PARP-1)), neovascularization (vascular endothelial growth factor (VEGF)), proliferation (antigen Ki-67 (Ki67)), and immune-cell infiltration including macrophages (F4/80 and CD163) and T cells (CD8 and CD4) (Figure 4A). Apoptotic pathways typically target proteins known as caspases. For instance, caspases 2, 8, and 9 are required for the initiation phase, and caspases 3, 6, and 7 are required for the execution phase of apoptosis [Parish 2013]. Exogenous H_2_S has been shown to down-regulate active caspase-3 in human esophageal squamous cell cancer cells, suggesting that H_2_S exerts anti-apoptotic effects in cancer cells [Lei 2016]. H_2_S has also been shown to activate PARP-1, an ADP-ribosylating enzyme and a component of the earliest response to DNA damage, suggesting that H_2_S may promote DNA repair [Zhao 2014]. The VEGF downstream signaling pathway has also been implicated in breast cancer cell growth, migration, and invasion by CSE, suggesting that H_2_S production promotes VEGF-led neovascularization in cancer [Wang 2019]. Ki67 is an important marker of bladder cancer prognosis, as high Ki67 levels are indicative of poor bladder cancer patient survival and recurrence [Ko 2017]. Finally, the tumor microenvironment evades anti-tumor immune responses through the recruitment of tumor-associated macrophages and favoring pro-tumor M2 TAM polarization, which in turn, dysregulates T-cell function [Noy 2014 and Mantovani 2004]. In mice, tumor-infiltrating TAMs generally express F4/80 and M2 TAMs and specifically express CD163, allowing quantification of total macrophage vs. M2 TAMs.

Caspase-9^+^ cells were preserved in all BBN^+^ groups compared to the BBN^+^ saline group (Figure 4B). PARP-1^+^ cells were induced in the BBN^+^ PAG, BBN^+^ GEM, and BBN^+^ PAG + GEM groups (*p* < 0.05, *p* < 0.0001, and *p* < 0.0002, respectively) but preserved in the BBN^+^ PAG + NaHS group compared to the BBN^+^ saline group (Figure 4C). PARP-1^+^ cells were further induced in the BBN^+^ PAG + GEM group compared to the BBN^+^ PAG group (*p* > 0.002, Figure 4G). Ki67^+^ cells were reduced in the BBN^+^ PAG, BBN^+^ GEM, and BBN^+^ PAG + GEM groups (*p* < 0.002, *p* < 0.002, and *p* < 0.0001, respectively) but preserved in the BBN^+^ PAG + NaHS group compared to the BBN^+^ saline group (Figure 4D). VEGF^+^ cells were reduced in the BBN^+^ PAG, BBN^+^ PAG + NaHS, BBN^+^ GEM, and BBN^+^ PAG + GEM groups compared to the BBN^+^ saline group (*p* < 0.0002, *p* < 0.05, *p* < 0.0002, and *p* < 0.0002, respectively; Figure 4E). Moreover, VEGF^+^ cells were further reduced in the BBN^+^ PAG + GEM group compared to the BBN^+^ PAG group (*p* < 0.05, Figure 4E). F4/80^+^ macrophages were induced in the BBN^+^ PAG group (*p* < 0.002) but preserved in all other BBN^+^ groups compared to the BBN^+^ saline group (Figure 4F). CD163^+^ macrophages were not altered in any group (Figure 4G). CD8^+^ T cells were induced in the BBN^+^ PAG and BBN^+^ PAG + GEM groups (*p* > 0.05 and *p* > 0.0001, respectively) but preserved in all other BBN^+^ groups compared to the BBN^+^ saline group (Figure 4H). Moreover, CD8^+^ T cells were further induced in the BBN^+^ PAG + GEM group compared to the BBN^+^ GEM group (*p* > 0.002, Figure 4H). CD4^+^ cells were reduced in the BBN^+^ PAG group (*p* < 0.05) but preserved in all other BBN^+^ groups compared to the BBN^+^ saline group (Figure 4I).

## 3. Discussion

In this study, the inhibition of endogenous H_2_S production reduced BC cell viability and tumor progression while enhancing the anti-cancer effects of chemotherapy. The gene expression of H_2_S-producing enzymes is downregulated in BC cells, yet positively correlates with the BC stage, suggesting that elevated H_2_S levels contribute to the aggressiveness of the cancer [22,23]. Under normoxic conditions, CSE is the most upregulated H_2_S-producing enzyme in BC cells [22]. Considering the hypoxic nature of the bladder tumor microenvironment [25], we reported CSE gene expression as the most upregulated over time, implying that, even under hypoxic conditions, CSE may be the most prominent H_2_S-producing enzyme in BC.

To investigate the effect of CSE-mediated H_2_S production on BC cell viability and bladder tumor progression, we inhibited CSE activity using the inhibitor PAG. We observed that PAG reduced cell viability, potentiated cell apoptosis, and attenuated tumor growth and invasion in vivo. Our findings support those of recent studies in which PAG reduced breast cancer cell viability, increased cell apoptosis [26], and decreased nasopharyngeal carcinoma tumor growth [27]. However, NaHS (H_2_S donor) reversed the effects of PAG, suggesting that inhibition of endogenous H_2_S production contributes to the observed anti-cancer effects of PAG. Furthermore, we investigated the effects of PAG in combination with GEM chemotherapy, a clinically used intravesical therapeutic agent for BC. PAG enhanced the cytotoxic effect of GEM by further reducing BC cell viability, inducing tumor regression and abrogating tumor invasion. This suggests a potential additive effect of PAG in the presence of chemotherapy. Our results are in line with the finding from a previous study in which combination therapy with PAG and cisplatin reduced BC cell viability compared to cisplatin alone and enhanced cisplatin cytotoxicity by further reducing tumor size [23].

To reveal the mechanism(s) underlying this synergistic effect, we stained bladder tumors for markers of apoptosis, proliferation, neovascularization, and immune-cell infiltration, as H_2_S promotes carcinogenesis through these pathways [16,28,29]. It was previously reported that PAG induces apoptosis through cleaved caspase-3 [23], which is directly cleaved by caspase-9. GEM has also been reported to induce apoptosis by activating caspase-9 [30]. Conversely, we found no significant change in caspase-9^+^ cell infiltration following monotherapy with PAG or GEM. Nevertheless, caspases also induce apoptosis by cleaving the DNA repair initiator, PARP-1. It has been reported that GEM promotes PARP-1 degradation [31], which we corroborated in the present study. In addition, we found that monotherapy with PAG-induced cleaved PARP-1^+^ cells. Unsurprisingly, combination therapy with PAG and GEM further increased PARP-1^+^ cells, suggesting that PAG enhances the pro-apoptotic effect of GEM through PARP-1 degradation. Furthermore, Ki67, a proliferative marker, is associated with a worse BC prognosis [32]. Khan et al. [26] reported a decrease in Ki67^+^ cells by PAG in human breast cancer xenograft tumors. We also observed a decrease in Ki67^+^ cells following monotherapy with PAG and GEM, with a further decrease following combination therapy, suggesting a further reduction in bladder tumor proliferation and a better prognosis. Neovascularization is another critical process in cancer progression, in which H_2_S is a key mediator [18]. Our group previously demonstrated reduced angiogenesis following the inhibition of endogenous H_2_S production in clear-cell renal cell carcinoma [15]. In this study, we observed a decrease in VEGF expression following monotherapy with PAG and GEM, and a further reduction after combination therapy, which poses another explanation for the additive effect of PAG during chemotherapy.

The immune system plays an important role in eliminating cancer cells. However, in many tumor microenvironments, anti-tumor immune responses are suppressed through the recruitment of tumor-associated macrophages (TAMs) [33], where pro-tumor M2 TAM polarization is favored [34], which in turn, dysregulates T-cell function. It has been reported that GEM has no significant effect on immune-cell infiltration, such as macrophages and T cells, within the bladder tumor [35], a finding that was corroborated in the present study. We observed that PAG potentiated F4/80^+^ macrophage infiltration alone and in conjunction with GEM, suggesting that PAG is capable of evoking an immune response. However, we found no significant changes for CD163^+^ M2 macrophages. Therefore, it is not possible to confirm with our findings as to what type of macrophage is abundant in this response. Nonetheless, other studies suggest that H_2_S reduces CD8^+^ T-cell infiltration in cancer [36]. Our study found an increase in CD8^+^ T-cell infiltration following monotherapy with PAG and a further increase after combination therapy with GEM, suggesting that PAG evokes an anti-tumor immune response. We also reported a decrease in CD4^+^ cell infiltration after monotherapy with PAG and combination therapy with GEM. CD4^+^ T cells comprise several subsets of immune cells, including immunosuppressive regulatory T cells, which are abundant in BC [28]. Remarkably, H_2_S depletion has also been shown to decrease regulatory T-cell infiltration in cancer [28]. Therefore, it is possible that inhibition of endogenous H_2_S production in our study may have depleted regulatory T cells from the bladder tumor. However, further investigations using FOXP3 are required to confirm this. Nonetheless, these findings suggest that the addition of an anti-tumor immune response by PAG may be partially responsible for the increased potency of GEM.

It is important to note that PAG inhibits many other pyridoxal 5′-phosphate-dependent enzymes, creating the potential for off-target effects of PAG administration. Many of these enzymes are involved in critical metabolic pathways. For instance, alanine transaminase is a PLP-dependent enzyme vital for amino acid metabolism and energy production and a biomarker of liver function. Therefore, it will be important to verify the presence, if any, of these off-target effects in the treatment of BC with PAG. However, the reversal of the effects of PAG by NaHS in this research strongly suggests that what we observed is due to CSE inhibition. It is also speculated that direct administration into the bladder may mitigate these off-target effects found in other organs. Further research will need to be conducted to support this.

Although our model does not completely reflect clinical practice, our observation of tumor regression and abrogation of invasion are promising for a novel BC therapeutic. Within the clinical setting, NMIBC is typically treated with transurethral resection of the bladder tumor followed by intravesical therapy. Therefore, the beneficial effect of PAG and GEM combination therapy that was observed in the absence of surgery may be even more beneficial when debulking surgery is performed. Moreover, our study demonstrated the immediate effectiveness of combination therapy, which in a clinical setting, is beneficial to patients who are unresponsive to conventional therapies. However, the long-term effectiveness of this combination therapy, as well as any potential side effects of H₂S inhibition, remains unknown. Therefore, future research should consider monitoring tumor growth for a longer period of time after treatment has stopped and should also investigate the potential side effects of H₂S inhibition. The risk of resistance is also important to consider. It is currently unknown whether BC cells can develop resistance to PAG treatment, and this knowledge is vital for the development of PAG as a BC therapeutic. Also, future studies should consider mechanistic details, such as signaling pathways or molecular targets specifically affected by the combination therapy used in our study. Finally, safety will also need to be evaluated. No immediate treatment side effects were observed in this study (i.e., hematuria or death). However, a deeper investigation into other side effects, such as damage to surrounding tissue, fatigue, nausea, etc., will require monitoring.

It is also important to note that we observed the development of MIBC. The gold-standard treatment for MIBC involves neoadjuvant chemotherapy followed by radical cystectomy. Intravesical therapy, as performed in this study, is typically reserved for NMIBC. However, as we observed tumor regression and abrogation of invasion following PAG and GEM combination therapy without any other therapeutic intervention, this may also be a promising treatment for MIBC patients.

## 4. Materials and Methods

### 4.1. Cell Culture and Reagents

Human BC cells (5637 cell line; provided by the Burton Lab., Western University, ON, Canada) were maintained in an RPMI 1640 medium containing 10% heat-inactivated fetal bovine serum and 1% penicillin/streptomycin and maintained under normal growth conditions of 21% O_2_ and 5% CO_2_ at 37 °C. NaHS (H_2_S donor) was purchased from Thermo Fisher Scientific (Burlington, ON, Canada). PAG (CSE inhibitor) and GEM (chemotherapeutic agent) were purchased from MilliporeSigma (Burlington, MA, USA).

### 4.2. Quantitative PCR (qPCR) Analysis

Seeded into 6-well plates were 5637 cells (2 × 10^5^ cells/well). After 24 h in normal culture conditions, cells were subjected to 0, 8, or 36 h of hypoxia (5% CO_2_, 0.5% O_2_, 95% N_2_) at 37 °C using a HypOxystation H85 hypoxia chamber (HYPO_2_YGEN, Frederick, MD, USA). These timeframes were selected, as they efficiently represent the progression of hypoxia. Cell lysate was homogenized, and total RNA was isolated using a QIAshredder and an RNeasy^®^ Mini Kit (Qiagen, Toronto, ON, Canada), respectively, and reverse transcribed into cDNA using a OneScript^®^ Plus cDNA synthesis Kit (ABM, Milton, ON, Canada) in conjunction with Oligo(dT)_12-18_ primers. The reaction mixture of each qPCR sample was prepared as per the BlastTaq^TM^ 2X qPCR MasterMix (ABM, Milton, ON, Canada) protocol and analyzed using a QuantStudio^TM^ 3 Real-Time PCR System (Thermo Fisher Scientific, Burlington, ON, Canada). The primer sequence for β-actin was designed using Primer-BLAST software version 1.1.0 (NCI), and primer sequences for CSE, CBS, and 3-MST were designed as previously described (Table 1) [22]. All genes of interest were normalized against β-actin. Fold changes in gene expression were compared to the no-hypoxia condition and were calculated using the ∆∆Ct method.

### 4.3. Flow Cytometry

Seeded in 6-well plates were 5637 cells (2 × 10^5^ cells/well). After 24 h in normal culture conditions, cells were subjected to 8 h of hypoxia at 37 °C. Cells were then washed with phosphate-buffered saline and treated with single (20 mM PAG, 100 μM NaHS or 100 μM GEM) or combination treatments (20 mM PAG and 100 μM NaHS or 20 mM PAG and 100 μM GEM) and subjected to 24 h of hypoxia. Cell viability was assessed by staining with Annexin V-FITC and propidium iodide (BioLegend, San Diego, CA, USA). Following this step, the cells were analyzed using CytoFLEX S V4-B2-Y4-R3 and CytExpert Software version 2.6 (Beckman Coulter, Brea, CA, USA).

### 4.4. Experimental Animals and Reagents

Six-week-old male C57BL/6 mice were purchased from Charles River Canada (St. Constant, QC, Canada) and maintained in the Animal Care and Veterinary Services facility at Western University (London, ON, Canada) under standard conditions. Animal studies were approved by the Animal Care Committee of the University Council on Animal Care (AUP Number: 2022-021). BBN (a carcinogenic agent) was purchased from TCI America (Portland, OR, USA).

### 4.5. Murine BC Model

Mice were randomized into BBN^+^ and BBN^−^ groups. The BBN^+^ mice received 0.05% BBN-supplemented tap water (BBN^+^) for 12 weeks to induce BC development, followed by access to untreated tap water for the remainder of the experiment. The BBN^−^ mice served as control and received saline for the full experimental duration.

### 4.6. Magnetic Resonance Imaging

To confirm the development of BC after BBN exposure, the mice underwent an MRI on week 14. All MRI examinations were acquired on a 3.0T GE MR750 clinical MR scanner (General Electric, Mississauga, ON, Canada) using a custom-built gradient and radio-frequency coils and under isoflurane anesthesia, during which the heart rate and body temperature were monitored. Bladder images were acquired using the GE system 3D steady-state free precession imaging sequence and fast imaging employing steady-state acquisition (FIESTA), and T2/T1 weighted images were produced. The scanning parameters were as follows: in-plane spatial resolution = 200 × 200 μm, repetition time = 6.5 ms, echo time = 2.3 ms, bandwidth = 31.25 kHz, flip angle = 35°, and scan time = approximately 36 minutes per mouse. The MRI images were analyzed using Horos imaging software, version 3.3.6. The total tumor burden was represented as the bladder wall volume, which was a modification from a previously described method [37]. Two regions of interest were manually segmented on the bladder, with one around the outer bladder wall and the second around the inner bladder lumen. The bladder wall volume was quantified as the volume difference. To evaluate tumor growth following intravesical therapy, the mice underwent a second scan on week 19. Tumor growth was calculated as the difference in bladder wall volume from the first to the second MRI.

### 4.7. Intravesical Therapy

The BBN^+^ mice were randomly assigned to an intravesical therapy group (*n* = 6 mice per group), where they received 80 μL of monotherapy (saline, 20 mM PAG, 100 μM NaHS, or 100 μM GEM) or 80 μL of combination therapy (20 mM PAG and 100 μM NaHS or 20 mM PAG and 100 μM GEM). The BBN^−^ cohort (*n* = 6) received saline. Therapies began on week 15 and were administered once every week for four weeks. A 26-gauge angiocatheter was inserted into the urethra under isoflurane anesthesia, and the treatments were delivered via intravesical administration.

### 4.8. Histological Staining

At the experimental endpoint (post-second MRI) bladders were harvested and placed in 10% neutral-buffered formalin for histological analysis. Four-micrometer-thick histological sections of the bladder were stained with hematoxylin and eosin and scored by a blinded genitourinary pathologist to assess cancer presence and level of invasion. IHC staining was performed by incubating the bladder sections with antibodies against the apoptotic markers caspase-9 and cleaved PARP-1, the neovascularization marker VEGF, the proliferation marker Ki67, and the macrophage markers F4/80 and CD163, as well as the T-cell markers CD8 and CD4 (Abcam, Toronto, ON, Canada). The sections were analyzed under a light microscope at 40× magnification, where five randomly selected fields were analyzed per section. Positive staining per field of view was quantified by Image J version 1.51 (National Institutes of Health, Bethesda, MD, USA).

### 4.9. Statistical Analysis

Data were analyzed via one-way or two-way ANOVA using Graphpad Prism statistical software package version 9.0 (LA Jolla, CA, USA) followed by Tukey’s post hoc test and expressed as mean ± standard error of the mean (SEM). Statistical significance was accepted at *p* < 0.05.

## 5. Conclusions

In conclusion, inhibition of endogenous H_2_S production reduced BC cell viability and tumor progression and enhanced GEM cytotoxicity, resulting in tumor regression and abrogating invasion. This effect was partly due to the role of PAG in enhancing the antineoplastic effects of GEM, as well as its immunogenic effect. Therefore, our finding suggests that H_2_S may be a novel target for developing improved BC diagnosis and treatments.

## Figures and Tables

**Table 1:** List of qPCR primer sequences.

Primer	Forward Sequence (5′→3′)	Reverse Sequence (5′→3′)
β-actin	AGCACAGAGCCTCGCCTTT	ATCATCATCCATGGTGAGCTGG
CSE	AGGTTTAGCAGCCACTGTAAC	GGGGTTTCGATCCAAACAAGC
CBS	GGCCAAGTGTGAGTTCTTCAA	GGCTCGATAATCGTGTCCCC
3-MST	CATTTCGCGGAGTACGCAG	GCTGGCGTCGTAGATCACG

## Data Availability

The original contributions presented in the study are included in the article.

## Supplementary Materials

The following supporting information can be downloaded at: https://www.mdpi.com/article/10.3390/ph17091212/s1, Figure S1: MRI images at 13 weeks. Column 1 (MRI 1) represents images after 12 weeks with BBN-treated water followed by 1 week with regular water. Column 2 (MRI 2) represents images at the end of intravesical therapy (19 weeks).
