# Supplementary material for: Pharmacological Inhibition of Endogenous Hydrogen Sulfide Production Slows Bladder Cancer Progression in an Intravesical Murine Model
Source: Pharmaceuticals (Basel). 2024 Sep 14;17(9):1212. doi: 10.3390/ph17091212 (PMC11435360; doi:10.3390/ph17091212)
Supplement: Supplementary file 1 [file pharmaceuticals-17-01212-s001.zip › pharmaceuticals-3161288-supplementary.pdf]

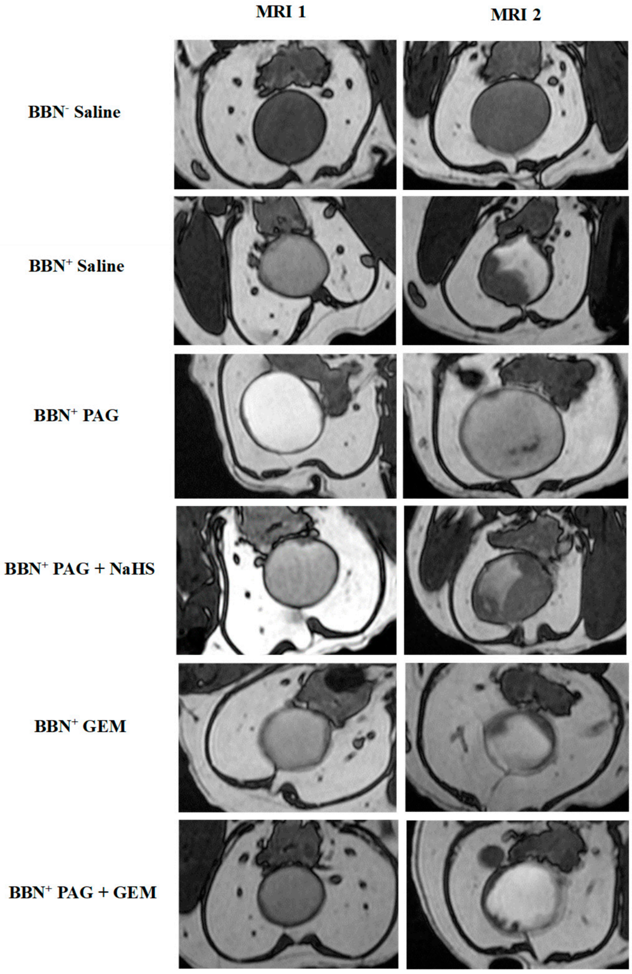

Figure S1. MRI images at 13 weeks. Column 1 (MRI 1) represents images after 12 weeks with BBN-treated water followed by 1 week with regular water. Column 2 (MRI 2) represents images at the end of intravesical therapy (19 weeks).
